# Lipid-Based Nanocarriers as Topical Drug Delivery Systems for Intraocular Diseases

**DOI:** 10.3390/pharmaceutics13050678

**Published:** 2021-05-09

**Authors:** Jose Navarro-Partida, Carlos Rodrigo Castro-Castaneda, Francisco J. Santa Cruz-Pavlovich, Luis Abraham Aceves-Franco, Tomer Ori Guy, Arturo Santos

**Affiliations:** 1 Tecnologico de Monterrey, Escuela de Medicina y Ciencias de la Salud, Campus Guadalajara, P.C., Zapopan 45138, Mexico; josenavarro@tec.mx (J.N.-P.); crodrigocastro@gmail.com (C.R.C.-C.); A01250093@itesm.mx (F.J.S.C.-P.); A00832512@itesm.mx (L.A.A.-F.); guytomer5@gmail.com (T.O.G.); 2 Centro de Retina Medica y Quirurgica, S.C., Centro Medico Puerta de Hierro, P.C., Zapopan 45116, Mexico

**Keywords:** ocular drug delivery, ocular barriers, lipid-based nanosystems

## Abstract

Effective drug delivery to intraocular tissues remains a great challenge due to complex anatomical and physiological barriers that selectively limit the entry of drugs into the eye. To overcome these challenges, frequent topical application and regular intravitreal injections are currently used to achieve the desired drug concentrations into the eye. However, the repetitive installation or recurrent injections may result in several side effects. Recent advancements in the field of nanoparticle-based drug delivery have demonstrated promising results for topical ophthalmic nanotherapies in the treatment of intraocular diseases. Studies have revealed that nanocarriers enhance the intraocular half-life and bioavailability of several therapies including proteins, peptides and genetic material. Amongst the array of nanoparticles available nowadays, lipid-based nanosystems have shown an increased efficiency and feasibility in topical formulations, making them an important target for constant and thorough research in both preclinical and clinical practice. In this review, we will cover the promising lipid-based nanocarriers used in topical ophthalmic formulations for intraocular drug delivery.

## 1. Introduction

Delivery of drugs to the intraocular tissues is one of the main concerns both pharmacists and ophthalmologists face every day. Over the past several years, conventional topical ophthalmic formulations such as eye drops, suspensions and ointments have been widely used in ophthalmic therapeutics, but none of them have demonstrated the sufficient capacity to increase the bioavailability to intraocular tissues without increasing the drug toxicity [1]. Consequently, to date, the use of invasive interventions such as intravitreal injections or intraocular implants are the main strategies to deliver drugs into the eye [1,2].

As it is well-known, intravitreal injections are the prototype intervention for the treatment of retinal diseases. Recently, intraocular biodegradable implants and micro pumps have been developed in order to deliver drugs into the eye. Although excellent results have been achieved with these devices, they are expensive and required to be implanted through a surgical procedure, limiting their availability to the population. Additionally, along with the intravitreal injections, they are not exempt from complications such as strabismus, infection or a conjunctival erosion related to the presence of an external device and postoperative complications including endophthalmitis or worsening of the visual acuity [2]. Therefore, the challenge to deliver drugs into the eye with increased bioavailability to either segment, and with fewer adverse events, is still unsolved.

All limitations related to the ocular delivery routes arise from the premise of the enormous complexity of the eye’s anatomy including its anatomical and physiological barriers. Nevertheless, at present, nanotechnology has been part of a new era, where new technological advances have made possible the creation of different nanosystems capable of overcoming those barriers [3].

Nanotechnology is defined as the development of materials on a 1 to 100 nm scale [4]. The usage of nanotechnology in the biomedical field had led to the creation of a hybrid science named nanobiotechnology [5], and with it, new systems of drug delivery arose and became known as nanosystems which act as drug nanocarriers [3]. These drug nanocarriers provide new opportunities to decrease the limitations of the conventional drugs, such as solubility, metabolic degradation, increased dosing frequency or lack of viable drug targeting [6,7,8]. The nanocarriers are classified into three main groups: polymeric, non-polymeric and lipid-based nanocarriers, which in turn subdivide into more specific groups [8]. The polymeric nanocarrier classification includes four main branches: the nanomicelles, the polymeric nanoparticles, dendrimers and nanogels. On the other hand, two nanomolecules, gold nanoparticles and mesoporous silica, are included for the non-polymeric nanocarrier group. Finally, lipid-based nanocarriers refer to the different subgroups including the emulsion-based, vesicle-based and particulate systems [8,9]. 

Recently, lipid-based nanocarriers have emerged as promising nanosystems for intraocular drug delivery. For example, topical liposome-based nanosystems have demonstrated, in preclinical and early clinical studies, to be efficient to deliver triamcinolone acetonide (TA) into the vitreous and retina [10,11]. This technological advance in the ophthalmic pharmacology field represents the opportunity to treat multiple inflammatory and neovascular intraocular disorders avoiding or reducing the use of intravitreal injections. Although intravitreal injection of TA is a well-described and capable route to release this steroid into the posterior pole of the eye, this procedure is not exempt from potential severe complications such as endophthalmitis, lens injury and retinal detachment [1,12,13]. These menaces related to the intravitreal injection of TA, are avoided by the topical instillation of topical liposome-based nanosystems. Moreover, the topical application allows to immediately suspend the effect of steroids by suspending the application (hours) [14], while in the intravitreal application the effect is maintained for long periods until the clearance is achieved (months) [15]. Therefore, in cases of adverse effects related to TA, the control in intravitreal-injected patients is complex.

In another example, the usage of nanoemulsions containing dorzolamide hydrochloride topically administered has proved to increase the therapeutic effect along with rapid and sustained action, thus decreasing intraocular pressure and demonstrating their effectiveness on the field [16].

Due to the potential clinical use of lipid-based nanocarriers in ophthalmology, we describe the characteristics of these nanosystems, as well as their principal targets on the ocular tissue. In order to achieve a better comprehension of the importance of the lipid-based nanocarriers in ocular drug delivery, in the first part of the text, we describe the anatomy of the ocular globe emphasizing the relevance of the ocular drug barriers to posteriorly deepen in the current knowledge about lipid-based nanosystems.

## 2. General Anatomy of the Ocular Globe

**Figure 1:**
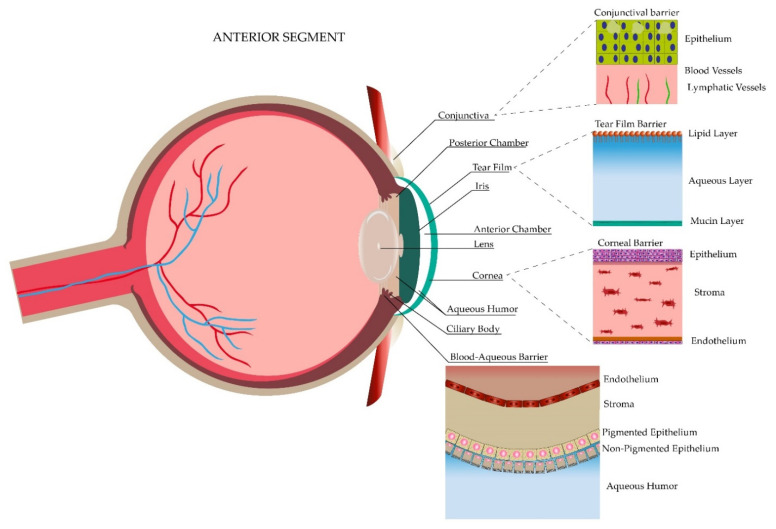
Anterior segment of the eye.

**Figure 2:**
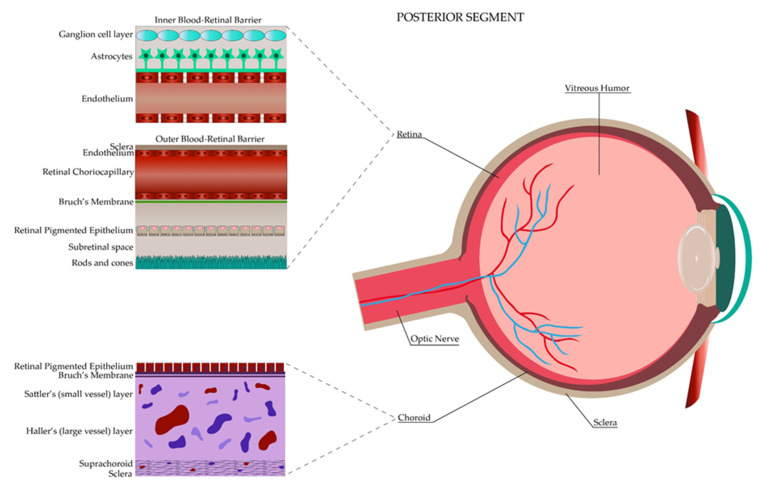
Posterior Segment of the eye.

The eye is classified into two main regions, the anterior segment and the posterior segment. The anterior segment represents the one-third frontal part of the eye and it is demarcated anteriorly by the cornea, laterally by the anterior drainage angle and posteriorly by the iris and lens. At the same time, this segment is divided into an anterior and posterior chamber. Each chamber is a fluid-filled cavity with aqueous humor produced by the ciliary processes of the ciliary body, which is in charge of nourishing the lens and keeping the intraocular pressure of this segment. Conversely, the posterior segment represents the resting two-thirds of the eye demarcated from the lens to the optic nerve, including specific structures such as the neural retina, retinal pigment epithelium, choroid, sclera and the vitreous humor, which provides nutrients to the lens and gives support to the retina [17,18,19,20,21]. Figure 1 and Figure 2 depict both regions along with the ocular barriers explained in the next section.

## 3. Ocular Drug Barriers

The particular anatomy and physiology of the eye form three protective barriers: static, dynamic and metabolic. These barriers represent a limitation for the drug administration to the eye. The hydrophilic and/or lipophilic properties, as well as different charges (cations or anions) of the drug molecules, will facilitate or impede their transport into the eye [22,23]. In the next section, we describe the characteristics of the ocular drug barriers.

### 3.1. Static Barriers

Corresponding to the anterior segment, the first static barrier protecting the eye is the cornea, which comprises 5% of the total ocular surface while the other 95% is conformed by the conjunctiva [24]. Ultrastructurally, it is composed of three main layers, the epithelial cells, the stroma and the endothelial cells. The corneal epithelium measures around 50 μm and is composed of four to six layers of non-keratinized stratified squamous epithelial cells [25]. This epithelium allows the passive transport of hydrophobic drugs smaller than 10 Å through the transcellular pathway [26]. Conversely, it blocks the passage of hydrophilic drugs that would enter through the paracellular pathway due to the presence of tight junctions (zonula occludens) [27,28]. The conjunctiva, which is the thin and transparent structure composed of a mucous epithelial, adenoids and fibrous layer covering one-third of the eyeball [29], increases the protective function and corresponds to the second static anterior barrier. One of its main functions is the formation of the tear film by producing electrolytes, fluid and mucins, but in contrast to the cornea, the conjunctiva has demonstrated lipophilic affinity to drugs [30] due to its increased paracellular spaces (230 times greater) and higher pore density (16 times higher) [31]. The third static barrier protecting the anterior segment is the blood–aqueous barrier supported by tight junctions (claudin 2 in the ciliary epithelium) between the non-pigmented epithelial cells (NPE), which is composed of cells of the endothelial lining of the iris [32]. As in the cornea, these junctions limit the diffusion of ions and small solutes through the paracellular space and separate the apical and basolateral domains of NPE cells, achieving a special configuration apical to apical of NPE and pigmented cells (PE). This configuration acquires an important role in mechanisms regulating the secretion of aqueous humor, which is possible through the ion transporters in those cells and the presence of gap junctions, composed mainly of connexin 43. The ions are transported through the basolateral membrane of PE cells, pass through the NPE cells via gap junctions and are finally released to the posterior chamber, generating an osmotic gradient and making water available for the aqueous humor formation. In this scenario, the modulation of tight junctions, by cytokines, for example, would have an impact on the absorption of different drugs [33]. Lastly, the efflux pumps are the fourth anterior static barrier, where the prototype proteins are the ATP-binding cassette (ABC) proteins, which help to transport substrates to the extracellular fluid. Because of their localization in the apical or basolateral membranes, these proteins can either enhance or restrict the drug bioavailability. The latter function is exerted by two main proteins, the P-glycoprotein 1 (P-gp), MDR1 or ABCB1 in the apical membrane, and the multidrug resistance proteins (MRP) or ABCC1 in the basolateral membrane. Each protein impedes the passage of either amphipathic compounds or organic anions, respectively. P-gp has the particularity of controlling the accumulation of drugs, especially by decreasing it either in healthy or diseased cells. Both proteins can be found in the iris, cornea, ciliary muscle and conjunctival epithelium [13,28].

The posterior segment has three different static barriers: the sclera, the brunch membrane, the blood–retinal barrier and efflux pumps. The sclera has four layers, being from the outermost to the innermost: the episclera, stroma, lamina fusca and epithelium [34]. Permeability is better through the sclera than in the anterior structures. It is dependent either on chemical properties of the substrate such as the size (radius, the most important), molecular weight, charges or lipophilicity, where the hydrophilic permeation is faster, or structural properties such as the presence of pores [13,35]. The Bruch’s membrane, which forms part of the five layers of the choroid, is a 2–4 µm membrane that helps in the prevention of hydrophilic compounds permeation and corresponds to the second static barrier [13]. The blood–retinal barrier (BRB) is different from the blood barrier of the anterior segment. It is composed of an outer and an inner BRB layer, both of which are supported by tight junctions where the former one presents them between the retinal pigment epithelium (RPE) and the fenestrated choriocapillaris, and the latter one between the non-fenestrated endothelium. Mainly, the BRB regulates the passage of solutes to the subretinal space and is in charge of maintaining retinal homeostasis. As in the sclera, hydrophilic components tend to pass through the paracellular route in comparison to the lipophilic ones [13,33,36]. Finally, the efflux pumps comprise the fourth static barrier. These pumps consist of the same two main proteins of the anterior segment explained previously, the P-gp and the MRP, which are located in the non-pigmented ciliary epithelium and the RPE [13].

### 3.2. Dynamic Barriers

Dynamic barriers refer to the physiological barriers which are in constant replacement and help protect the eye by clearing molecules through different mechanisms [37]. Four dynamic barriers have been described; three in the anterior segment: (1) tear film, tear turnover and drainage, (2) conjunctival lymph and blood flow, and (3) aqueous humor and one in the posterior segment: the choroidal lymph and blood circulation.

The tear film is composed of three layers: a superficial lipidic layer of 0.1 μm thickness, which prevents the evaporation of the aqueous layer; a middle aqueous layer of 8 μm thickness, allowing the spread of tears in the eye surface; and an inner mucous layer of 0.8 μL composed of mucin that enables the adhesion of the aqueous layer. The mucous layer creates a hydrophilic gel layer clearing pathogens and restricting drug delivery [38,39]. At the same time, an alteration in tear pH triggers a stimulus for initiating its production, contributing to the wasting of the drug, hence the importance of maintaining a pH between 7 and 7.7 [38]. Additionally, blinking and tear turnover represent two important aspects to consider. Blinking creates a pump that allows the lacrimal fluid to be distributed throughout the eye surface and then transported into the nasolacrimal duct [39]. This flow and production of lacrimal fluid allow the administered drug to be in contact with the eye surface for approximately 1–2 min. In relation to topical drug administration, it has to be considered that the reflex blinking consists of rapid consecutive blinks, each of 0.1 s of duration that decreases the ability of the eye to hold transiently 30 μL [12,39]. On average, a person blinks at a rate of 15–20 blinks per minute with a tear volume of about 7–9 μL, with a basal tear turnover of 0.5–2.2 μL per minute [40] (mean of 1.2 μL/min) [38]. Although less information has been gathered, the second dynamic barrier refers to the conjunctival blood and lymphatic vessels, which act in consonance to maintain the metabolic function and immune protection offered to the anterior compartment in different inflammation scenarios such as the presence of a foreign body [41]. Furthermore, the last anterior dynamic barrier, which is a crystal fluid known as the aqueous humor, is produced by the ciliary body in the pars plicata where the ciliary processes are found. Besides having an impact in determining the intraocular pressure, the aqueous humor is responsible for protecting the avascular structures of the anterior chamber, such as the lens and the cornea, by removing waste metabolic products and providing the necessary nutrition to maintain ocular homeostasis. It represents an easy pathway of drug distribution for the anterior segment [18,42]. On the contrary, the posterior segment dynamic barrier is given only by the choroidal circulation. The choroid is composed of an outer or Haller’s layer and an inner or Sattler’s layer, where large and small vessels are the main components. Choroidal vessels are responsible for 85% of the eye’s perfusion and its high flow system has demonstrated an important role in drug clearance by decreasing the concentration of the ocular administered hydrophilic drugs [43,44].

### 3.3. Metabolic Barriers

In spite of being able to overcome all of the previous physiological barriers, metabolism represents the last barrier that could intervene with the correct effect of ocular drugs. Elimination of medications occurs mainly through metabolism by conjugating or oxidizing the substance in place. Enzymes of the cytochrome P450 family, peptidases or esterases, are present in ocular tissues, giving them the ability to avoid drug accumulation and also to convert prodrugs into active drugs modifying the solubility, bioavailability and concentration [45]. Table 1 summarizes all the ocular barriers along with their main functions.

## 4. Ocular Drug Delivery Routes

**Figure 3:**
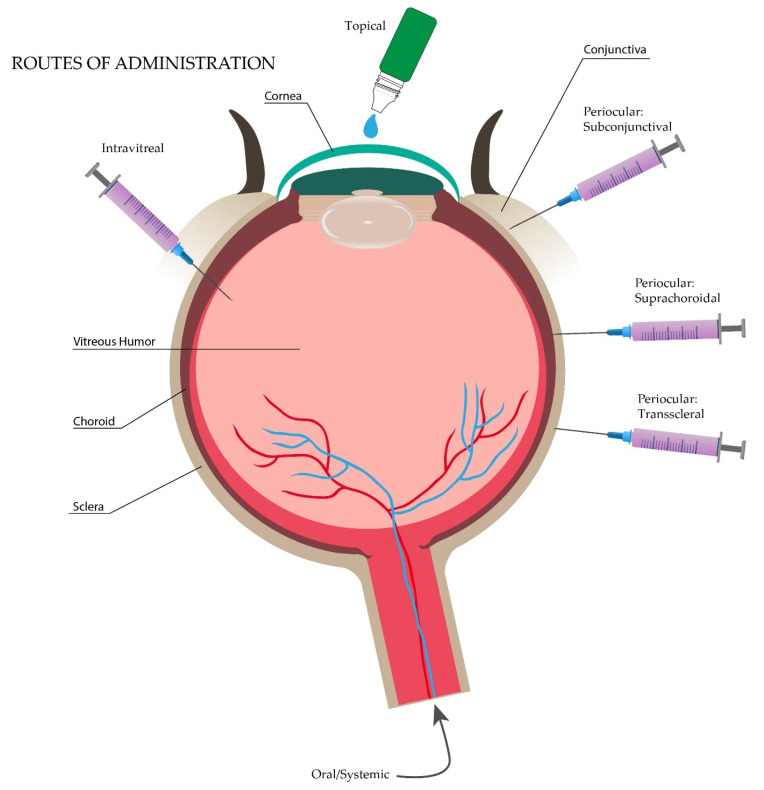
Ocular routes of administration.

There are several acute and chronic diseases that can affect the anterior as well as the posterior segment of the eye [46]. Worldwide, approximately 253 million people are affected with some type of visual impairment, from which 38.5 million are estimated to be classified as blind [47,48]. For treatment of these diseases, there are several routes for drug administration schematized in Figure 3, including topical, oral/systemic, periocular, and intravitreal injections, and the best choice depends on the target ocular tissue [49]. As mentioned before, besides the drug administration pathway, the pharmacokinetics of drug diffusion across the ocular barriers is dependent on the molecular dimensions, molecular weight, atomic charge, and chemical components of the drug [22]. Hydrophilic compounds tend to permeate through the stroma of the cornea and the sclera more rapidly than lipophilic (hydrophobic) molecules, making the delivery of lipid-dominant molecules such as corticosteroids more challenging [35,50]. On the other hand, corneal epithelium, and retinal pigment epithelium (RPE) have lipophilic properties that allow the easy penetration of these compounds [26,35].

Instillation of drugs over the ocular surface (topical route) is the method of preference for the treatment of ocular diseases, specifically for the anterior segment, because it represents an easy and noninvasive method of administration with demonstrated better patient compliance [1,12,24]. Eye drops cover around 90% of the ophthalmic formulations. However, because of the presence of the dynamic barriers in the precorneal area such as tear turnover or nasolacrimal drainage system, the average drug volume present after topical administration is around 35 to 56 μL. This volume is reduced to only 25 μL because of the conjunctival sac capacity when the lower eyelid is pulled away and is reduced even more (10 μL) when it returns to its normal form. Therefore, although this route is used frequently, these limitations decrease the bioavailability in the anterior and posterior segments to less than 5%. Improvement in new ocular drugs in terms of bioavailability and permeation, such as viscosity and permeation enhancers, or cyclodextrins, have revolutionized some aspects of these concerns. Even then, they present disadvantages in drug loss [1,24].

Attending the inadequate bioavailability of the posterior segment through topical administration, intravitreal injections have represented the most recommended route for treating the posterior ocular diseases [1,24]. However, patient compliance is affected by its invasiveness and the adverse effects that the procedure entails, including inflammation and infection (endophthalmitis), retinal hemorrhage or detachment, increased intraocular pressure, among others [1,12,13]. In addition, intravitreal injections are expensive due to the requirement for monthly dosing, frequent hospital visits, and associated after-care costs [51].

Oral or systemic administration represents a less recommended route for drug delivery to intraocular tissues because of the related systemic effects. This route involves drug delivery through systemic circulation, crossing all the way to the blood–ocular barriers, including the blood–aqueous barrier and the blood–retinal barrier. Although the extent of the drug to the retina could be improved through this route, the presence of the blood–ocular barriers offers a rigorous permeability regulation in drug delivery to this area, presenting an approximate bioavailability of 2% to the intraocular tissue [12,22,52].

Lastly, the periocular route has its own subdivisions, the most important ones being subconjunctival, which allows escaping conjunctival vessels used for elimination but is limiting for water-soluble drugs; the suprachoroidal, which minimize systemic and intravitreal adverse effects passing only through the choroid; and the transscleral, which mainly surpasses the anterior segment [12,22].

Every route of administration has its own limitations either for the anterior or posterior segment of the eye. The primary challenge of ocular drug delivery is to circumvent the superficial ocular tissues in order to achieve therapeutically effective concentrations of drugs in the intraocular tissues [53]. Currently, many research groups in academia and industry are focused on developing novel formulations to overcome these barriers [54]. Nanoparticulated systems could be the answer to overcome these limitations because of their higher bioavailability, better absorption and reduced side effects [49,53].

## 5. Topical Nanosystems for Intraocular Drug Delivery

**Figure 4:**
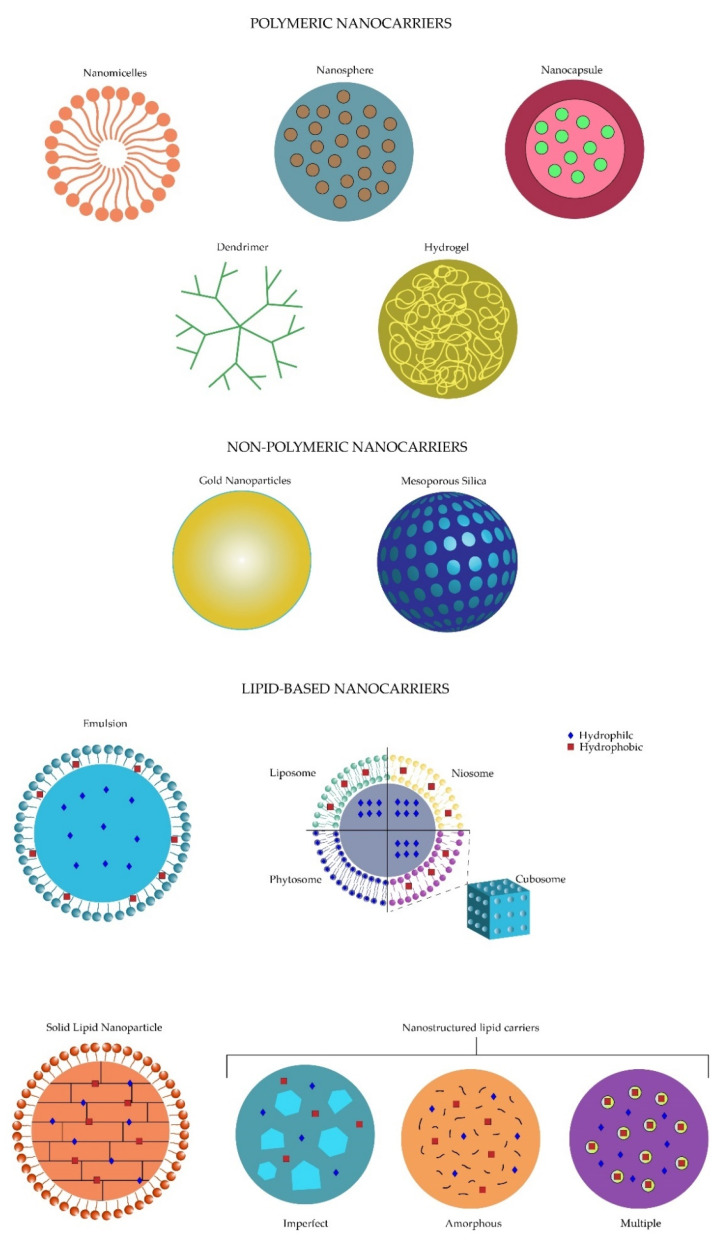
Structure of the different topical nanosystems.

As mentioned before, the topical route represents the most manageable approach considering its major advantages, such as patient treatment adherence, easy, non-invasive administration, low cost, availability in the market [22] and the decreased incidence of complications [20]. Ointments, emulsions and suspensions comprise the different forms of conventional formulations used in the present day with delivery supremacy in terms of bioavailability, solubility and increased residence time in the precorneal area. Currently, nanotechnology is embracing those formulations to overcome the defects still present [1,12,24]. Different polymeric, non-polymeric and lipid-based nanosystems have been proposed to be administered topically in order to deliver drugs into the eye. Figure 4 demonstrates the structure and characteristics of the different topical nanosystems, specifying drug integration for the lipid-based nanocarriers.

### 5.1. Topical Polymeric Nanosystems for Intraocular Drug Delivery

Nanomicelles are one of the topical ophthalmic polymeric nanosystems which have been demonstrated to be promising (Figure 4). These nanostructures, measuring 20–200 nm, contain polymers that are self-assembled and embedded in an aqueous solution. Nanomicelles present with an hydrophobic core and a hydrophilic shell composed of poly (ethylene) glycol (PEG), which makes them an amphiphilic molecule for drug transport [55,56]. Although they are non-biodegradable and immunological reactions could exist regarding their composition, these formulations tend to offer better stability, along with a sustained release and less toxicity profile. Recently, Xu et al. have demonstrated their functionality after using a nanomicelle with chitosan oligosaccharide carrying dexamethasone administered topically and showing the same residence time in the precorneal area and delivery efficiency to the posterior segment [57].

Polymeric nanoparticles are a second example of topical ophthalmic promising polymeric nanocarriers. These formulations, which include nanocapsules or nanospheres (Figure 4), range from 10–100 nm composed of proteins, lipids and synthetic polymers. The low cost, increased time stability, non-toxic characteristics and biodegradability of the polymeric nanoparticles demonstrate a more advantageous profile than nanomicelles [58]. A nanosphere containing ciclosporin A and applied through topical administration demonstrated significant results regarding the corneal (6–8 times higher) and conjunctival concentration when compared to the control group, supporting the effectiveness of these formulations [59]. 

Dendrimers are another example of nanotechnology applied to the development of topical ophthalmic formulations for intraocular drug delivery. These are distinguished from the other polymeric molecules in their particular macromolecular branched structure surrounding the core (Figure 4), which gives them the ability to increase water solubility, encapsulation and dispersity. Both hydrophobic and hydrophilic drugs are capable of being included in these formulations [60,61]. Adverse effects when they are used topically, including blurred vision or a veil formation that would progressively end in vision loss, have been demonstrated when applying dendrimer formulations [60,62]. Yang et al. implemented a codelivery drug topical administration using dendrimers carrying brimonidine and timolol maleate for glaucoma treatment and showed higher concentrations in different ocular tissues such as the cornea, conjunctiva and the aqueous humor, along with a sustained reduction of the intraocular pressure in rabbits. In this study, no adverse effects were recorded [63].

Lastly, nanogels are implemented for topical ophthalmic formulations. Nanogels are characterized by a molecular morphology of a cross-linked polymeric sphere, which confers the ability to transport both hydrophilic and hydrophobic drugs (Figure 4). A polymer, either natural, synthetic or both, can be used for the composition of its structure [64,65]. Differing from the nanomicelles, these formulations are not immunogenic and are biodegradable. Nanogels present the particularity of being in a liquid state at low temperatures but become a gel after contacting the eye; hence their name [66]. Disadvantages arise due to their poor colloidal stability and aqueous solubility and the rapid elimination by the macrophages [65]. Nanogels have been studied by Mohammed et al. using a formulation carrying fluconazole to treat fungal infections in the cornea, and demonstrated a greater bioavailability and penetration through corneal tissue, as well as an increased antifungal activity, showing another possible carrier for efficient ocular delivery [67].

### 5.2. Topical Non-Polymeric Nanosystems for Intraocular Drug Delivery

Non-polymeric nanocarriers include gold nanoparticles (Figure 4) comprised of a metallic nanoparticle, which have demonstrated a wide variety of applications in ophthalmology additionally to drug delivery, including anti-angiogenesis, imaging, gene therapy, biosensing, brachytherapy, among others [68]. Even though gold nanoparticles have presented an easy production and a biocompatible profile [69,70,71], their accumulation in dendritic cells, as well as in macrophages, stem cells or endothelial cells, have shown to alter their cytokine secretion, where this accumulation induces oxidative stress, leading to cytotoxicity [72,73,74]. Several studies have shown the utility and promising carriage of different drugs into gold nanoparticles to increase penetration and bioavailability to deeper ocular tissues [68,75,76]. Cho et al. evaluated in vivo mice the use of these nanoparticles in corneal neovascularization and obtained a significant reduction (39.8%) of it. Gold nanoparticles demonstrated their anti-inflammatory and anti-angiogenic effect by inhibiting the ERK pathway [77].

Mesoporous silica also forms part of the non-polymeric nanocarriers. These nanoparticles use silicates polymerized with surfactants in an aqueous solution. Mesoporous silica structure is composed of 2–6 nm pores organized in a solid framework (Figure 4) [78,79], giving them the capability to carry a high concentration of drugs. Additionally, mesoporous silica are non-toxic, biocompatible, and biodegradable, and have shown to decrease intraocular pressure and an increased bioavailability after being topically administered carrying brimonidine for glaucoma treatment [80].

### 5.3. Lipid-Based Nanosystems for Intraocular Drug Delivery

Although the existence of a wide variety of nanosystems, lipid-based nanosystems have shown an increased efficiency over the last years, making them a target for constant and thorough research. Moreover, lipid-based nanosystems have already been studied in a preclinical and phase I clinical trial with promising results [11], increasing their importance for further study. For this reason, this review will describe the characteristics of the most important lipid-based nanocarriers (Figure 4), as well as their principal targets on the ocular tissue.

## 6. Topical Lipid-Based Nanosystems for Intraocular Drug Delivery

Lipids are water-insoluble, organic compounds chemically composed of molecules with polar hydrophilic heads and nonpolar hydrophobic tails. Although lipids usually present hydrophobic properties, the presence of lipids with amphipathic behavior has also been shown [81]. Currently, lipid qualities have been demonstrated by functioning as necessary vehicles in ocular drug delivery. These advantages are conceded by their specific properties regarding melting points, crystallinity and ability to be polymorphic.

Positive outcomes on the implementation of lipid-based nanocarriers include the formulation flexibility and design for administration, increase in bioavailability, solubility and permeation, benefit–risk scale inclined to an almost no-hazard result; and a more regulated release of the ocular drug [8].

Lipid-based nanosystems are divided into three main groups: emulsion-based, vesicle-based and particulate systems, which present their own composition and benefits in drug delivery systems [9].

### 6.1. Emulsion-Based

Emulsions are used mainly in two forms, oil-in-water (Figure 4) or the other way around, where the former one is the best choice for the ocular tissues because of less mild adverse effects such as irritation. The main objective of these formulations is the increase of drug residence time in the precorneal area, as well as improving the permeation through the cornea to achieve a better bioavailability [1]. Liu et al. reported a comparison of azithromycin in aqueous solutions and lipid emulsions, where an increased conjunctival drug residence time, sustained release and an improved bioavailability were demonstrated by the emulsions [82]. Another study performed in vivo mice by Lin Ying et al. evaluating lipid emulsions using marked eye drops with fluorescence showed an increased intensity on the retina using these formulations [83]. Subgrouping of the emulsion-based nanosystems gives rise to micro and nanoemulsions. Microemulsions are isotropic, thermodynamically stable droplets, sized from 20–200 nm, compared to the 1–20 µm emulsion size, based on oil, water, and surfactant composition, which is formed spontaneously [8,84,85]. Oil-in-water type formulation offers the droplets a dispersed configuration in a continuous aqueous phase, compared to the water-in-oil type which is dispersed in the continuous oil phase [84,86]. The oil component in the microemulsions plays an important role either in their curvature or in an increased penetration enhancing property. In addition, microemulsions have the ability to reduce interfacial and surface tension, resulting in an increase of the permeability of both hydrophilic and lipophilic drugs [87] through the corneal surface by adding surfactants and co-surfactants, such as alcohol or salts, to maintain thermodynamic stability [84,88,89]. Although these formulations present a high probability of mild adverse effects, including irritation, their simple preparation, increased drug solubility, stability and bioavailability with the consequent rise in their efficacy, parallel to the low-cost production, represent the major advantages of these compounds [8,90].

Even though nanoemulsions share similar characteristics with microemulsions, including the size of 10–200 nm, an oil/water formulation combined with an emulsifier in less quantities such as the surfactant, proteins or lipids, they have presented differences in terms of thermodynamic instability, kinetic stability and the way they are obtained: by mechanical force [8,85,91]. The usage of nanoemulsions has been demonstrated to increase solubility and bioavailability and reduce the frequency of drug dosing along with a sustained and constant release of the drug due to their interactions with the lipid layer conforming the tear film. More importantly, these formulations are planned to be the replacement for liposomes and conventional eye formulations after showing more stability, faster action with a smaller dose, and the capability to transport hydrophilic and lipophilic drugs [8,85,91,92]. Major limitations fall back to the high-cost production and the need for advanced equipment, as well as a reduced time of stability arising from the fact of using less surfactant.

### 6.2. Vesicle-Based

Another type of lipid-based nanosystem is the vesicle-based nanocarriers, including a wide variety of subgroups such as liposomes, niosomes, cubosomes and phytosomes/herbosomes mainly used for ocular tissues, pharmacosomes implemented for the gastrointestinal tract, ethosomes and archaeosomes for skin and systemic circulation, among many others [8]. Liposomes were first described in 1965 by Alec Bangham [93], but it was not until the early 1970s when Gregory Gregoriadis started researching drug delivery applications of liposomes [94]. The word liposome is derived from the Greek “lipo”, referring to their fatty content, and “soma”, referring to their structure [95]. Liposomes are self-assembling colloidal vesicles composed of a phospholipid and cholesterol bilayer with an encapsulation property (Figure 4) [96]. These microscopic vesicles, with varying sizes from 10 nm to 1000 nm or greater, contain an internal aqueous volume surrounded by an amphipathic lipid bilayer. The phospholipid component of the liposome membrane contains either a natural molecule, including phosphatidylethanolamine, phosphatidylglycerol, phosphatidylcholine, phosphatidylserine, and phosphatidylinositol [96], or a synthetic one created via organic chemistry or enzymatic synthesis processes [97]. Depending on their structure, liposomes can be classified as unilamellar vesicles (ULVs) and multilamellar vesicles (MLVs) [96]. Moreover, ULVs can be subclassified depending on their size as small unilamellar vesicles (SUVs, 20–200 nm), large unilamellar vesicles (LUVs, 200–1000 nm), and giant unilamellar vesicles (GUVs, >1000 nm). Additionally, MLVs generally measure greater than 500 nm and consist of more than one lipid bilayer separated by an aqueous compartment [98]. After Gregory Gregoriadis’ research of liposomes as a potential ocular drug delivery system [94], four interaction mechanisms between liposomes and cellular membranes were described, including adsorption, endocytosis, fusion and lipid exchange. Adsorption contributes to a better drug uptake by promoting passive diffusion. Once the liposomes are adsorbed into the cell membrane, the liposome becomes leaky due to the surface proteins of the membrane, thus increasing the proximity drug concentration of the cell membrane and drug uptake [98]. After adsorption, the liposome can be engulfed and internalized, a process known as endocytosis. Lysosomes will fuse to endosomes, with the subsequent degradation of liposome lipids and release of the drug to the cytoplasm. Nonetheless, loss of drug may occur due to enzymatic degradation by those same lysosomal enzymes [98,99]. The third interaction mechanism is fusion with the cell membrane and releasing the drug directly into the cytoplasm [98,100]. Lastly, due to their shared characteristics, the lipids of both structures can be transferred, destabilizing the liposome and consequently releasing the drug [98,101].

Liposomes can transport hydrophilic, hydrophobic, and amphipathic drugs, with a greater improvement when using a lipophilic drug [102,103]. These formulations are biodegradable with a relatively non-toxic behavior, which enhances drug permeation by binding to the corneal surface [102]. A wide variety of liposome preparation methods exist, where thin-film hydration, consisting of the use of an organic solvent to dissolve lipid molecules after evaporation and film rehydration, has become the most recognized [104]. Even though liposomes may appear to be an excellent delivery system, conventional liposomes suffer challenges that limit their usage. Instability represents one of their main limitations due to the fact that liposome unsaturated lipids can be oxidized or hydrolyzed, and to leakage of the entrapped drug that alters their structure [98]. Stability also relates to the present alkyl chains, where the long chains tend to demonstrate a lower permeability than short chains [105]. Furthermore, liposomes can aggregate and fuse into bigger particles, which could trigger an immune response and render them unable to be absorbed by the ocular tissue [106]. Lastly, conventional liposomes do not differentiate between target and non-target cells, making targeted drug delivery an issue. All these limitations have been explored to be surpassed by the implementation of lyophilization to achieve higher stability or the use of bioadhesive polymers, such as chitosan and collagen, to prolong corneal residence time [107,108,109,110]. Additionally, charged liposomes have been developed to overcome aggregation and fusion, where positively charged liposomes have demonstrated to be more efficient at increasing drug delivery than neutrally or negatively charged ones, as they can interact with the negatively charged cornea tissue and increase their residence time [111]. Regarding drug leakage, strategies such as increasing the cholesterol amount in the lipidic bilayer have been implemented and proven effective [111,112,113].

Liposomes can be administered in almost all routes of administration. As previously explained, absorption is enhanced when using positively charged liposomes and was demonstrated by Law et al. using acyclovir [111]. Chetoni et al. used these charged liposomes on topical formulations and showed better outcomes in terms of bioavailability compared with conventional formulations [114]. Another study by Yan Shen and Juasheng Tu showed 2 to 10 times more concentration distributed in ocular tissues, especially on vitreous humor, sclera, lens, iris and cornea using ganciclovir in a liposome formulation [115]. Santos et al. have demonstrated the stability of a topical Triamcinolone Acetonide Liposome Formulation (TALF) and showed better concentrations of Triamcinolone Acetonide vitreous and retina, with the highest peak at 12 h after topical administration of an eye drop [14]. In addition, this research group reported a significant clinical improvement with no adverse events after the administration of topical instillation of TALF for 90 days in two pilot studies including 12 human eyes with macular edema secondary to branch retinal vein occlusion and 12 with refractory pseudophakic cystoid macular edema [10,116]. Furthermore, liposomes have shown better outcomes using intravitreal injections (IVTs) compared with conventional solutions. Regarding the onset and progression of cataracts, Zhang et al. applied IVTs using cationic liposomes carrying cytochrome c to rats, exhibiting a more significant effect than a conventional cytochrome c solution [117]. Moreover, Natarajan et al. compared topical latanoprost with a loaded liposome injection of latanoprost in rabbits and demonstrated a decrease in the intraocular pressure for up to 90 days [118]. Lastly, in terms of the subconjunctival route, liposome formulations have shown promising results reaching the retinal epithelium and have been reported by Kaiser et al. applying a subconjunctival injection of a liposome carrying minocycline with a successful outcome [119]. The liposome variability and application in clinical practice previously presented demonstrates the effectiveness of these formulations in different ocular diseases and offers several options to preserve patient compliance and better curative outcomes regarding the possibility of different administration routes.

The similarity between liposomes and niosomes is evident. Niosomes are vesicular, surfactant-based molecules that surpass liposomes in terms of toxicity or stability [120]. Niosomes are also classified based on their size and the number of layers including MLVs and ULVs with their subgrouping [121]. Differing from the phospholipid layer of the liposomes, niosomes are composed of a nonionic surfactant, such as ether or ester-linked or di-alkyl chain which are single chain, added to cholesterol, which makes them non-toxic, improves the mechanical rigidity of the vesicle, reduces the leakiness of the membrane, and increases its entrapment efficiency (Figure 4) [8,121,122]. Niosomes can transport either hydrophilic or hydrophobic drugs through almost all delivery routes and have demonstrated improved stability, permeation, and bioavailability compared to liposomes. An enhanced penetration is achieved by the inhibition of the epithelial cells P-gp and opening of tight junctions. In addition, because of its unique property of not having any charge, niosomes become more compatible and less immunogenic [8,53,120]. The main disadvantages of niosomes include hydrolyzation and leakage of the drug. Bhardwag et al. reported in a review the ability of niosomes combined with polyethyleneglycol (PEG) to surpass the phagocytic system and, as a result, increasing drug residence time in the circulation [120]. The use of niosomes should be recognized as a substitute for conventional ocular treatments due to the nontoxicity that they present, making them more manageable regarding the dosage.

Cubosomes, another example of vesicle-based nanocarriers, and measuring around 100–300 nm, are crystal-clear liquid molecules formed by amphiphilic lipids in the cubic phase and a stabilizer or surfactant, such as F127 or monoglyceride glycerol monoolein, which are self-assembled, offering them the ability to accommodate hydrophilic and hydrophobic drugs and their characteristic morphology (Figure 4). Cubosomes present two water channels conforming to their structure that will be bicontinuous and separated by the lipid bilayer. Compatibility to corneal epithelial cell membrane structure enables the fusing of the cubosomes and creates a drug reservoir for ocular tissues. Similar to niosomes, cubosomes present increased stability compared to liposomes, as well as an enhanced penetration of the drug, which is achieved by the inhibition of the epithelial cells P-gp and opening of tight junctions [8,123,124,125]. Improvements with the use of cubosome formulations have been demonstrated in treating glaucoma by Li et al. compared with traditional eye drops in rabbit corneas, where an increase of two times on transcorneal flux was observed using poloxamer 407 and glycerol monoolein combined with pilocarpine nitrate [124]. In addition, an increased residence time, as well as a reduced intraocular pressure (50%), was observed after the instillation of these formulations. Han et al. also reported better bioavailability and fewer adverse effects when combining cubosomes with flurbiprofen and dexamethasone [8]. Even though cubosomes transport both hydrophilic and hydrophobic drugs, most of the studies point to the treatment of anterior segment diseases and less is described regarding the posterior segment.

Along with the previous vesicle-based nanosystems, phytosomes or herbosomes have recently demonstrated an important role in the area of ophthalmology [126]. These formulations have shown a similar structure to a cell which is composed mainly by the association of a phospholipid, such as phosphatidylcholine, with a plant [10,126,127]. This characteristic structure involving chemical bonds allows the phytosomes to form a complex when combined with the drug in a 1:1 or 2:1 ratio compared to the liposomes containing the hydrophilic drug in their water-soluble cavity with a definite ratio (Figure 4). Consequently, due to their increased drug absorption, phytosomes demonstrate better bioavailability [127]. A single study has been made of phytosomes in relation to ophthalmology, where they were used carrying L-carnosine and compared to *N*-acetyl-l-carnosine, its prodrug, in the cataractogenesis scenario and showed a significant inhibition and delayed progression of the lens opacification [126].

### 6.3. Particulate Systems

Particulate systems are a third option involving lipid particles in their formulations. Solid lipid nanoparticles (SLNs) refer to delivery systems measuring from 50–1000 nm. They are composed of a lipidical nucleus combined with an amphiphilic surfactant for stabilization, where either a hydrophilic or hydrophobic drug can be transported [8,123,128]. At room temperature, the lipids conforming these structures, including steroids, mono, di and triglycerides, or fatty acids, are solid, and represent 0.1–30% of their composition, which are dispersed in an aqueous solution (Figure 4) [128,129]. Specific characteristics of the SLNs, which provide them with several advantages, include stability, an increase in drug load and controlled release, protection against chemical degradation, increased safety and non-toxic effects by using physiological lipids. Moreover, SLN’s easy preparation and low cost give the opportunity for high-scale production. SLNs improve ocular drug delivery by enhancing the corneal absorption with better bioavailability, lengthening the drug retention time and maintaining a sustained release [8,92,123,129]. Five drug-releasing methods have been described including desorption, diffusion either through the matrix of the nanoparticle or the wall of nanocapsules, erosion, or a combination of erosion and diffusion [130]. The implementation of these formulations has been demonstrated by different authors. Regarding topical administration, Singht et al. established an increase in corneal and conjunctival uptake of isoniazid SLN formulation that was labeled with fluorescein [131]. Additionally, improvement in corneal permeability and bioavailability to posterior tissues was shown by Hippalgaonkar et al. when using an SLN with indomethacin compared with a conventional indomethacin solution [132]. Kakkar et al. also demonstrated the use of SLN to the posterior segment of the eye [133]. The research group reported in vivo rabbits an SLN carrying ketoconazol with significant favorable outcomes of antifungal potential on the posterior segment of the eye. Conversely, intravitreal administration has also been studied. Abrishami et al. used an SLN with diclofenac in rabbits and demonstrated a higher concentration by 2.5 and 6.5 times more in vitreous and aqueous humor respectively compared with the traditional drug [134]. Moreover, a study made by Pozo-Rodriguez et al. showed an increase in ocular retinal delivery combining protamine and an SLN-DNA non-viral vector [130,135]. These studies have shown the possibilities of implementing these formulations in clinical practice and the fact of achieving both anterior and posterior segments make them as equally important as the previous explained formulations.

Since its development in the early 1990s, nanostructured lipid carriers (NLCs) have shown a controlled nano-structuring of the lipid matrix due to the inclusion of a solid lipid with incompatible liquids, meaning solid plus a liquid lipid (Figure 4). NLCs can be prepared by high shear homogenization and ultrasound, high pressure (hot and cold homogenization), solvent emulsification/evaporation, and the microemulsion-based approach [128]. The composition of NLCs offers increased drug incorporation, including hydrophilic and lipophilic drugs, stability, and release properties, abilities that make NLCs the new SLNs generation [8,92,123,128,136,137,138]. NLCs can be divided into three types: the amorphous, the multiple structure and the imperfect (Figure 4). When a structureless matrix is created when mixing a solid lipid with a special one including hydroxy-octacosanyl, hydroxystearate or iso-propyl myristate it is classified as the amorphous type. The amorphous NLCs demonstrate the ability to prevent a premature drug expulsion caused by beta-modification due to the absence of an ordered state. On the other hand, the multiple structure NLCs are composed of several compartments of liquid oil in a solid matrix which increases the solubility and, consequently, the loading of the drug employed. Lastly, the imperfect type consists of the mixture of the fatty acids conforming several lipids and a carbon chain, creating imperfections in the crystal structure which are directly proportional to the drug loading capacity for lipophilic drugs [138,139]. NLCs formulations have demonstrated effectiveness in ocular drug delivery for both eye segments. For instance, Zhang et al. have demonstrated an increase in cellular uptake of the lens, and therefore an increase in the antiproliferative effect of an NLC carrying genistein formulation coated by chitosan hydrochlorides, similar to the enhanced transcorneal penetration (2.4 fold increase) outcome reported by Luo et al. when using an NLC chitosan-coated formulation [140,141]. Topical instillation NLC has been reported by Liu et al. showing almost a six-fold increase in the bioavailability of the aqueous humor when compared to the conventional drug solution [142]. Using this same route of administration involving an NLC loaded with triamcinolone acetonide, Araujo et al. demonstrated in vivo mice the ability to deliver lipophilic actives to the posterior segment of the eye via the corneal and non-corneal pathways [136]. Nanostructured lipid carrier characteristics including easy production, better stability, avoidance of using organic solvents, and sterilization feasibility make these formulations considerable for the pharmacological treatment of eye diseases [143,144]. Table 2 compares the lipid-based nanocarriers implemented either in preclinical or clinical trials for the anterior and posterior segment.

## 7. Conclusions

Lipid-based nanosystems have shown promising outcomes in relation to the actual challenges of ocular drug delivery, along with preserving the best care of the patients. To date, the main concern in ophthalmologic diseases falls back to the reduced bioavailability and an increased occurrence of adverse events. Furthermore, patient compliance due to the possible complications associated with non-topical routes of administration and economic solvency becomes an extra burden to overcome the different ocular diseases. As previously explained, topical ocular drug administration becomes the ideal non-invasive administration route due to the advantages offered, but still needs improvement regarding bioavailability. In our actual technologic decade, these problems have become more likely to be overcome with nanomedicine in our hands. Nanotechnology has given ophthalmologists and pharmacists the necessary tools to achieve these improvements using nanomolecules in order to increase ocular drug delivery with fewer adverse effects. Positive outcomes on the implementation of lipid-based nanocarriers have provided new opportunities to reduce the actual limitations including bioavailability, solubility, permeation, metabolic degradation, increased adverse effects and dosing frequency, or lack of viable drug targeting [6,7,8]. The increasing efficiency of lipid-based nanocarriers over the last years has made them a target for constant and thorough research. In fact, these formulations have been already used in both preclinical and clinical practice demonstrating favorable outcomes treating ocular diseases [10,11,16,51,87,91,98,103,126,131,145,146,147,148,149,150,151,152,153,154,155,156,157,158,159,160,161,162,163,164,165,166,167,168,169]. With all these promising results and taking advantage of the new technologies, researchers, pharmacists, and clinical professionals must continue this line of investigation and encourage the use of these new formulations in the near future in clinical medical practice.

## Figures and Tables

**Table 1:** Summary of the different ocular barriers and their main functions.

Segment of the Eye	Ocular Drug Barriers		Main Functions
Anterior	Static	Cornea	Allows passive transport of hydrophobic drugs and blocks transport of hydrophilic drugs.
		Conjunctiva	Formation of tear film, lipophilic affinity to drugs due to its paracellular spaces.
		Blood Aqueous Barrier	Limitation of ion and small solute diffusion and hydrophilic drugs. Regulation of aqueous humor secretion through ion transporters and gap junctions, connexin 43.
		Efflux pumps (ABC proteins)	Enhance or restrict transportation of substrates and drug bioavailability, including both hydrophilic and hydrophobic.
	Dynamic	Tear film, turnover, and drainage	Mucous layer creates a hydrophilic gel layer clearing pathogens and restricting drug delivery, especially hydrophobic drugs. Production and flow of lacrimal fluid avoids the accumulation of drugs.
		Conjunctival lymph and blood flow	Maintain metabolic function and immune protection.
		Aqueous humor	Protects avascular structures of the anterior chamber and provides nutrition to maintain ocular homeostasis. Easy drug distribution pathway for the anterior segment.
		Choroidal lymph and blood circulation	Responsible for 85% of the eye’s perfusion and helps in drug clearance by decreasing the concentration of hydrophilic drugs.
Posterior	Static	Sclera	Permeation dependent on chemical or structural properties. Allows a more rapidly hydrophilic permeation than hydrophobic.
		Bruch’s membrane	Helps in the prevention of hydrophilic compounds permeation.
		Blood Retinal Barrier	Regulates the passage of solutes to the subretinal space. Presents with hydrophilic permeation.
		Efflux pumps	Enhance or restrict transportation of substrates and drug bioavailability, including both hydrophilic and hydrophobic.
	Dynamic	Choroidal lymph and blood circulation	Responsible for 85% of the eye’s perfusion and helps in drug clearance by decreasing the concentration of hydrophilic drugs.
Anterior and Posterior	Metabolic	Cytochrome P450	Metabolize substances by conjugation or oxidation to avoid drug accumulation. Or convert prodrugs into active drugs modifying the solubility, bioavailability, and concentration.
		Enzymes: peptidases, esterases	

**Table 2:** Comparative table of lipid-based nanocarriers for the anterior and posterior segment.

Segment of the Eye	Disease	Lipid Nanocarrier	Development Stage	Description	Main Findings	Reference
Anterior	Uveitis	Emulsion-based	Preclinical study: rabbit	Microemulsions containing dexamethasone coated with chitosan or microemulsion with prednisolone administered topically.	Increased residence time in the precorneal area, better bioavailability and enhanced anti-inflammatory effect.	[103,145]
	Glaucoma	Emulsion-based	Preclinical study: rabbit	Microemulsions with timolol maleate or pilocarpine hydrochloride administered topically.	Better bioavailability, increased retention, and maintained or increased drug efficacy (reduction of IOP).	[87,146,147]
	Bacterial Keratitis	Emulsion-based	Preclinical study: rabbit	Microemulsions containing ofloxacin, gatifloxacin or moxifloxacin administered topically.	Sustained release, increased ocular concentration and therapeutic efficacy.	[87,148,149,150]
	Inflammatory diseases	Emulsion-based	Preclinical study: rabbit	Microemulsions with tacrolimus topically administered.	Enhanced penetration and concentration, sustained release.	[87,151]
Anterior	Glaucoma	Emulsion-based	Preclinical study: rabbit	Nanoemulsion with dorzolamide hydrochloride, y travoprost, or acetazolamide after topical administration.	Increased therapeutic effect with a rapid and sustained action, enhanced absorption.	[16,152,153]
	Bacterial Conjunctivitis	Emulsion-based	Preclinical study: rabbit	Nanoemulsion carrying moxifloxacin or besifloxacin administered topically.	Increased concentration and bioavailability, better efficacy and decreased dose application.	[91,154]
	Dry eye	Emulsion-based	Clinical trial	Nanoemulsion with Povidone-iodine topically administered.	Better therapeutic efficacy and improvement of symptoms.	[155]
	Inflammatory anterior ocular diseases	Emulsion-based	Preclinical study: rabbit	Nanoemulsion with tacrolimus for topical treatment.	Increased residence precorneal time and better bioavailability.	[156]
Anterior	Keratitis	Vesicle-based	Preclinical study: rabbit	Liposomes containing acyclovir, ganciclovir, tobramycin or fluconazole administered topically.	Increased bioavailability half-life of the drug and therapeutic efficacy, and better permeation.	[98]
	Glaucoma	Vesicle-based	Preclinical study: rabbit	Liposome with pilocarpine, latanoprost or acetazolamide administered topically.	Better entrapment, increased and sustained therapeutic effect and duration of action.	[98]
Posterior	Refractory macular edema	Vesicle-based	Preclinical study: rabbit Phase I clinical trial	Liposome carrying triamcinolone acetonide topically administered.	Improved permeation to the posterior segment (vitreous and retina) and therapeutic effect.	[10,11]
	Age-related macular degeneration	Vesicle-based	Preclinical study: rabbit and rat	Liposome with Bevacizumab administed topically.	Enhanced delivery and increased concentration (vitreous and retina).	[51]
	Choroidal neovascularization secondary to laser use	Vesicle-based	Preclinical study: mice	Liposome with diclofenac administered topically.	Enhanced permeability to posterior segment and therapeutic efficacy.	[157]
Anterior	Fungal keratitis	Vesicle-based	Preclinical study: rabbit	Niosome with natamycin plus ketorolac tromethamine administered topically.	Increased corneal infiltration and a higher level in the hypopyon.	[158]
	Glaucoma	Vesicle-based	Preclinical study:	Niosome containing timolol maleate topically administered.	Increased and maintained concentration in the aqueous humor.	[159]
	Conjunctivitis	Vesicle-based	Preclinical study: rabbits	Niosome with lomefloxacin HCl administered topically.	Improvement in penetration and therapeutic efficacy.	[160]
Anterior	Uveitis	Vesicle- based	Preclinical study: rabbit	Cubosome containing beclomethasone dipropionate administered topically.	Increased permeation through the corneal tissue with a better anti-inflammatory effect and tolerability.	[161]
	Glaucoma	Vesicle-based	Preclinical study: rabbit	Cubosomes with timolol maleate administered topically	Increased residence time, penetration and therapeutic effect.	[162]
	Keratomycosis	Vesicle based	Preclinical study: mice	Cubosome containing fluconazole topically administered.	Enhanced therapeutic effect.	[163]
	Cataracts	Vesicle-based	Ex vivo study: pig	Phytosome carrying L-carnosine.	Longer residence time and better therapeutic effect.	[126]
Anterior	Fungal keratitis	Particulate system	Ex vivo study: goat	SLN with Natamycin.	Sustained release with better permeation and increased therapeutic effect.	[164]
	Tuberculosis	Particulate system	Ex vivo: pig	SLN with isoniazid.	Improved corneal permeation.	[131]
	Keratitis	Particulate system	Preclinical study: rabbit	SLN containing tobramycin administered topically.	Increased bioavailability and drug retention.	[165]
Anterior and Posterior	Posterior ocular diseases (Diabetic macular edema, inflammation, uveitis)	Particulate system	Preclinical study: rabbit	SLN with triamcinolone acetonide administered topically.	Increased corneal permeation and residence time, higher concentration on both, aqueous and vitreous humor (sustained release).	[166]
Anterior	Anterior diseases	Particulate system	Ex vivo study: rabbit	NLC loaded with curcumin.	Enhanced permeability.	[167]
Posterior	Diabetic retinopathy	Particulate system	Preclinical study: mice	NLC with palmitoylethanolamide administered topically.	Increased retinal concentration and therapeutic efficacy.	[168]
Anterior and Posterior	Fungal infections (keratomycosis)	Particulate system	Preclinical study: rabbit	NLC loaded with amphotericin B administered topically.	Increased therapeutic effect and higher bioavailability in anterior and posterior ocular tissues.	[169]

## Data Availability

The data presented in this study are available on request from the corresponding author.
